# RDX Induces Aberrant Expression of MicroRNAs in Mouse Brain and Liver

**DOI:** 10.1289/ehp.11841

**Published:** 2008-09-19

**Authors:** Baohong Zhang, Xiaoping Pan

**Affiliations:** 1 Department of Biology, East Carolina University, Greenville, North Carolina, USA; 2 Department of Chemistry, Western Illinois University, Macomb, Illinois, USA

**Keywords:** carcinogenesis, gene regulation, microarray, microRNA, miRNA, qRT-PCR, RDX, toxicant, toxicity, toxicogenomics

## Abstract

**Background:**

Although microRNAs (miRNAs) have been found to play an important role in many biological and metabolic processes, their functions in animal response to environmental toxicant exposure are largely unknown.

**Objectives:**

We used hexahydro-1,3,5-trinitro-1,3,5-triazine (RDX), a common environmental contaminant, as a toxicant stressor to investigate toxicant-induced changes in miRNA expression in B6C3F1 mice and the potential mechanism of RDX-induced toxic action.

**Methods:**

B6C3F1 mice were fed diets with or without 5 mg/kg RDX for 28 days. After the feeding trials, we isolated RNAs from both brain and liver tissues and analyzed the expression profiles of 567 known mouse miRNAs using microarray and quantitative real-time polymerase chain reaction technologies.

**Results:**

RDX exposure induced significant changes in miRNA expression profiles. A total of 113 miRNAs, belonging to 75 families, showed significantly altered expression patterns after RDX exposure. Of the 113 miRNAs, 10 were significantly up-regulated and 3 were significantly down-regulated (*p* < 0.01) in both mouse brain and liver. Many miRNAs had tissue-specific responses to RDX exposure. Specifically, expression of seven miRNAs was up-regulated in the brain but down-regulated in the liver or up-regulated in the liver but down-regulated in the brain (*p* < 0.01). Many aberrantly expressed miRNAs were related to various cancers, toxicant-metabolizing enzymes, and neurotoxicity. We found a significant up-regulation of oncogenic miRNAs and a significant down-regulation of tumor-suppressing miRNAs, which included let-7, miR-17-92, miR-10b, miR-15, miR-16, miR-26, and miR-181.

**Conclusions:**

Environmental toxicant exposure alters the expression of a suite of miRNAs.

Hexahydro-1,3,5-trinitro-1,3,5-triazine (RDX, also known as hexogen or cyclonite) is a common environmental pollutant resulting from military and civil activities. According to the U.S. Department of Defense, an estimated 12,000 sites across the United States have been contaminated with explosives, including RDX. Concentrations of RDX in soils exceed thousands of milligrams per kilogram (Jenkins et al. 2006). RDX and its metabolites were also identified in water sources, including groundwater (Beller and Tiemeier 2002).

Environmental contamination by RDX and its *N*-nitroso metabolites has raised health concerns about human and environmental exposure (Zhang et al. 2008). It has long been known that RDX exposure causes neurotoxicity, immunotoxicity, and an increased likelihood of cancers. A causal relationship has been established between seizures and acute occupational RDX exposure in humans, with results confirmed in laboratory animals (Burdette et al. 1988; Pan et al. 2007; Testud et al. 1996). One early study demonstrated that RDX exposure elevated the incidence of tumors in B6C3F1 mice (Lish et al. 1984). Based on these findings, RDX has been classified as a class C potential human carcinogen by the U.S. Environmental Protection Agency (U.S. EPA 1988). Despite evidence supporting the role of RDX as a cytotoxic agent and potential chemical carcinogen, the molecular mechanism of RDX-induced neurotoxicity and potential carcinogenesis remains unknown.

Recently identified microRNAs (miRNAs) may play an important role in RDX exposure and in the process of RDX-induced tumorigenesis and neurotoxicity. miRNAs are a group of small non-protein-coding endogenous RNAs that posttranscriptionally regulate the expression of > 30% of human protein-coding genes (Lewis et al. 2005). miRNAs negatively regulate gene expression through translation inhibition, mRNA cleavage, or deadenylation/decap-mediated mRNA decay (Giraldez et al. 2006; Wu et al. 2006). These mechanisms are likely governed by the degree of complementarity between mRNAs and their targeting miRNAs (Zhang et al. 2007b). In animals, most miRNAs bind to their target mRNAs imperfectly for repressing protein translation at multiple complementary sites within the 3k untranslated regions (UTRs) (Ambros 2001), with a few exemptions at the open reading frames or 5k UTRs (Lytle et al. 2007). Many miRNAs are evolutionarily conserved in animals, from worms to humans (Pasquinelli et al. 2000), suggesting that miRNA functions are conserved from species to species.

miRNAs play an important role in almost all fundamental biological and metabolic processes in eukaryotic organisms (Zhang et al. 2007b). Many recent studies have demonstrated that miRNAs regulate cancer development, including cancer invasiveness and metastasis. Most commonly occurring cancers are associated with the aberrant expression of at least one miRNA (Zhang et al. 2007a). Moreover, it has been established that specific cancers have their unique miRNA expression profiles (Lu et al. 2005), suggesting that miRNAs respond to different cancers in different ways. Thus, miRNA profiles are useful for classifying and identifying cancers. Changes in miRNA expression can regulate the cancer development cascade. A recent *in vitro* study showed that transient overexpression of the miRNA let-7 in A549 lung adenocarcinoma cell lines inhibited lung cancer cell proliferation (Takamizawa et al. 2004). Another study found that changes in the expression levels of a single miRNA (miR-10b) could initiate tumor invasion and metastasis (Ma et al. 2007).

It is well known that many chemical toxicants and biological toxins cause different types of cancers. Although various environmental carcinogens cause DNA and chromosome damage as well as the aberrant expressions of cancer-related genes and toxicant-metabolizing enzymes, the pathogenic mechanisms of toxicant/toxin-induced cancers are unclear (de Kok et al. 2005; Myllynen et al. 2007). Because miRNAs play important roles in cancer development, and the aberrant expression of miRNAs has been observed in different cancers, we hypothesized that the exposure to a specific environmental procarcinogen, such as RDX, would induce alterations in miRNA expression, and that the altered miRNA expression contributes to carcinogenesis. To test this hypothesis, we exposed B6C3F1 mice to RDX and investigated the effect of RDX exposure on the global expression profile of miRNAs, particularly on the oncogenic, tumor-suppressing, and disease-related miRNAs. We assayed RDX-induced changes in miRNAs using microarray and quantitative real-time polymerase chain reaction (qRT-PCR) technologies. Given that miRNAs are highly conserved between mice and humans, the results would help us better understand the molecular mechanisms of RDX-related diseases, including potential carcinogenic and neurologic damages.

## Materials and Methods

### Animal treatment

We purchased female, virgin, B6C3F1 mice (*Mus muscaris*), at 9–11 weeks of age, from Charles River Laboratories, Inc. (Wilmington, MA). Mice were acclimated to the laboratory environment for 5 days before administering dietary RDX. Twenty-one mice were randomly assigned to treatment or control groups. Each cage housed three mice, and all cages were located in an animal room with temperature ranging from 68°F to 72°F and 25–75% relative humidity with 16/8-hr light/dark cycle. RDX-spiked food was used to feed mice and tap water was provided *ad libitum*, and their behavior and the weight of food and drinking water were carefully monitored twice per day. Animal use and handling protocols complied with Texas Tech University Animal Use and Care Committee guidelines. We treated all mice humanely and with regard for alleviation of suffering.

The mice in treatment groups were fed using finely ground Purina Certified Rodent Chow No. 5002 (Purina Mills, St. Louis, MO) containing 5 mg/kg RDX. RDX was dissolved in acetone and then sprayed onto the rodent chow to produce a dosage of 5 mg/kg. For the control group, we sprayed the same amount of acetone used in the RDX food preparation onto the rodent chow as a vehicle control. We thoroughly mixed the RDX-treated mouse food for at least 30 min after spraying with the acetone-solubilized solution and then spread it in a well-ventilated area to allow the acetone to evaporate for 4 days before use. The actual amount of RDX in the rodent chow was measured using pressurized liquid extraction followed by gas chromatography with electron-capture detection (Pan et al. 2005).

### Mouse euthanasia and organ collection

After 28 days of exposure, we euthanized both the RDX-fed and control mice by CO_2_ asphyxiation. The brain and liver tissues were immediately removed, weighed, and transferred into a 2-mL microcentrifuge tube and stored in liquid nitrogen during necropsy. We then transferred the frozen tissue samples into a −80°C freezer until miRNA analysis.

### RNA isolation

We extracted total RNA from each sample (brain and liver) using the mirVana miRNA Isolation Kit (Ambion, Austin, TX) according to the manufacturer’s instructions. Briefly, 0.03–0.05 g tissues were weighed and placed in a new 2-mL micro-centrifuge tube, followed by adding 300 μL lysis/binding buffer. Then, the tissues were thoroughly disrupted and homogenized using a Sonic Dismembrator (model 100, Fisher Scientific, Atlanta, GA). After homogenization, we added 30 μL miRNA homogenate additive to each tissue lysate, vortexed it for 10 sec, and then incubated it on ice for 10 min. After washing, we eluted the total RNAs using 100 μL elution buffer provided in the miRNA isolation kit. We performed all these operations on ice. The extracted RNA was quantified using a NanoDrop ND-100 spectrophotometer (NanoDrop Technologies, Wilmington, DE), aliquoted, and immediately stored at −80°C until analysis.

### miRNA microarray

The miRNA micro-array analysis was performed by LC Sciences (Houston, TX). Briefly, the assay started with approximately 6 μg total RNA. After the total RNAs were fractionated by size using a YM-100 Microcon centrifugal filter (Millipore, Billerica, MA), poly(A) tails were added to RNA sequences with lengths less than 300 nucleotides using poly(A) polymerase. Then, an oligonucleotide tag was ligated to the poly(A) tail for later fluorescent dye staining. RNA samples from liver and brain extracts were hybridized overnight using two different tags on a μParaflo microfluidic chip using a microcirculation pump developed by Atactic Technologies (Houston, TX) (Gao et al. 2004). One of the major advantages of the μParaflo microfluidic technology is that it allows efficient parallel synthesis of a large number of different oligonucleotide molecules (Sun et al. 2008). Each micro-fluidic chip contained the following probes: *a*) detection probes, which consisted of all chemically modified nucleotide sequences complementary to all 567 mouse miRNAs listed in the Sanger miRNA miRBase database (Release 10.1: December 2007; http://www.microrna.sanger.ac.uk/sequences/); *b*) a total of 49 positive and negative control probes designed by LC Sciences to ensure uniformity of sample labeling and assay conditions; and *c*) a spacer segment of polyethylene glycol to extend the coding segment away from the substrate. LC Sciences made the probes *in situ* using the photogenerated reagent chemistry. The melting temperatures of hybridization were balanced by chemical modifications of the probes, and hybridization reactions were performed in 100 μL 6× SSPE buffer (0.90 M NaCl, 60 mM Na_2_HPO_4_, 6 mM EDTA, pH 6.8) containing 25% formamide at 34°C. After RNA hybridization, tag-conjugating Cy3 and Cy5 dyes were circulated to control and treatment samples, respectively, for dye staining. Each analyzed miRNA was repeated six times and the controls were repeated for 4–16 times. A GenePix 4000B (Molecular Devices, Union City, CA) laser scanner was used to collect the fluorescence images, which were digitized using Array-Pro image analysis software (Media Cybernetics, Bethesda, MD).

### miRNA microarray data analysis

We analyzed miRNA microarray data by subtracting the background and then normalizing the signals using a LOWESS filter (locally weighted regression), as described in a previous report (Bolstad et al. 2003). We listed a miRNA as a detectable miRNA when it met at least three criteria: *a*) signal intensity greater than three times background standard deviation; *b*) spot coefficient of variance (CV) < 0.5, in which we calculated CV as (standard deviation)/(signal intensity); and *c*) at least three of the six repeats have signal greater than three times background standard deviation. The ratio of the two sets (control and treatment) of detected signals (log_2_ transformed, balanced) and *p*-values of the *t*-test were calculated. Differentially detected signals were those with *p*-values less than 0.01.

The detailed method for microarray data analysis and normalization is described in the Supplemental Material (http://www.ehponline.org/members/2008/11841/suppl.pdf (Bolstad et al. 2003).

### RT-PCR and qRT-PCR

We selected miRNAs with aberrant expression in micro-array analysis and then validated them using qRT-PCR on an ABI7300 system (Applied Biosystems, Foster City, CA). We used TaqMan miRNA assays to detect and quantify mouse miRNAs using stem-loop RT-PCR according to the manufacturer’s instructions. We provide a detailed description of the method in the Supplemental Material (http://www.ehponline.org/members/2008/11841/suppl.pdf).

### Prediction of miRNA targets

We used two different computational programs, TargetScan (Lewis et al. 2003) and PicTar (Krek et al. 2005), to predict miRNA targets. Each predicted gene was assigned a score from 1 to *n* according to the gene’s original score generated by each computational program. Then, the two scores from both programs were added up to give a total score for each predicted gene. The lower the total score a predicted gene has, the more likely it is the real target gene of a specific miRNA. We identified the 50 most promising candidate genes based on the additive scores. For details, see the Supplemental Material (http://www.ehponline.org/members/2008/11841/suppl.pdf).

## Results

### miRNA expression in mouse brain and liver tissues

**Figure 1:**
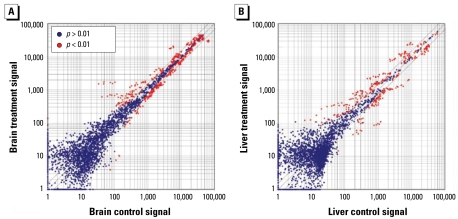
miRNA microarray signal distribution between control samples and RDX-exposed samples in mouse brain tissues (*A*) and liver tissues (*B*).

We observed differentially expressed patterns of miRNAs in mouse brain and liver. In this study, 369 miRNAs were detected in mouse brain and/or liver out of the 567 currently known mouse miRNAs (Figure 1). Of the 369 detected miRNAs, 284 were detected in the liver and 326 in the brain. Among detected miRNAs, 20 miRNAs were highly expressed in the liver, and 53 miRNAs were highly expressed in the brain [see Supplemental Material, Tables 1–3 (http://www.ehponline.org/members/2008/11841/suppl.pdf)]. Fifteen miRNAs were highly expressed in both liver and brain: miR-709, let-7a, let-7f, let-7c, let-7d, miR-26a, let-7b, let-7g, miR-26b, miR-29a, miR-126-3p, miR-23b, miR-30c, miR-16, and miR-23a.

**Figure 2:**
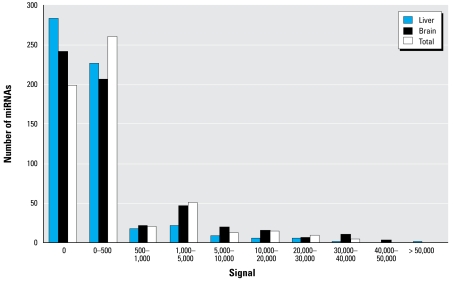
Signal distribution of all analyzed miRNAs in mouse liver and brain tissues by microarray assay.

The expression levels were low for most of the detected miRNAs. Of the 369 detected miRNAs, 269 gave signals lower than 500 (Figures 1 and 2). However, 89 miRNAs were expressed with signals greater than 1,000; some of them with signals greater than 40,000. This suggests that the expression levels of miRNAs in mice vary greatly, with some produced at only a few copies per cell and others present in hundreds of copies. This observation is similar to that of a previous report (Lagos-Quintana et al. 2002).

miRNA expression patterns significantly differ between mouse liver and brain. Some miRNAs were highly expressed in the brain but not in the liver, and vice versa [see Supplemental Material, Tables 1–3 (http://www.ehponline.org/members/2008/11841/suppl.pdf)]. In this study, 85 miRNAs were expressed in the brain only. Some of these miRNAs, for example, miR-9, miR-9*, miR-218, miR-204, and miR-129-3p, are highly expressed in the brain with signals higher than 40,000. However, we did not detect these miRNAs in the liver, suggesting that these miRNAs are specific to brain relative to liver. Except for the brain-specific miRNAs, the expression levels of 68 miRNAs in the brain were at least 10-fold higher than those in the liver. On the contrary, 43 miRNAs were expressed in the liver but not in the brain, suggesting that these miRNAs are liver specific compared with brain. In addition to the 43 liver-specific miRNAs, five miRNAs (miR-148a, miR-192, miR-194, miR-122, and miR-21) were highly expressed in the liver but at low levels in the brain; four of them (miR-148a, miR-192, miR-194, miR-122) were expressed at levels at least 10-fold greater in the liver than in the brain. miR-10a and the miR-689 were also expressed in the liver at levels 10-fold higher than those in the brain[see Supplemental Material, Table 2 (http://www.ehponline.org/members/2008/11841/suppl.pdf)].

### RDX exposure altered miRNA expression profiles

**Figure 3:**
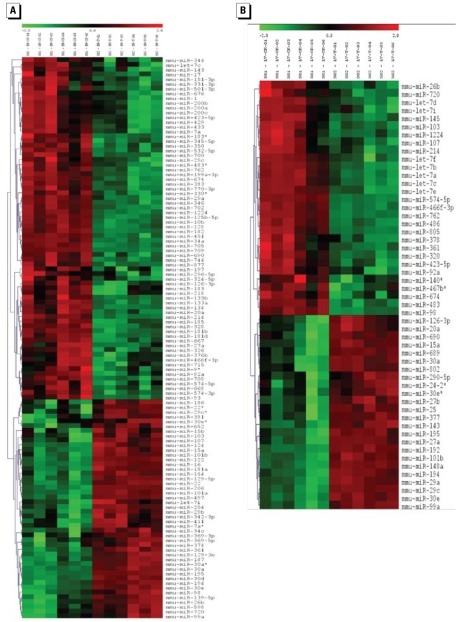
Hierarchical clustering of the differentially expressed miRNAs in control and RDX-treated mouse brain (*A*) and liver (*B*) samples after exposure to 5 mg/kg RDX for 28 days. Each row represents one miRNA with significantly differential expressions between control and treatment (*p* < 0.01). Each column represents a biological replicates; in each panel, the left six columns are for controls and the right six for RDX treatments. Colors represent expression levels of each individual miRNA: red, up-regulation; green, down-regulation.

A comparison of miRNA expression levels between control and RDX-treated mice revealed that RDX exposure significantly altered miRNA expression profiles [Figure 3; see Supplemental Material, Table 1–3 (http://www.ehponline.org/members/2008/11841/suppl.pdf)]. RDX exposure resulted in the expression of a greater number of miRNAs in brain tissues from RDX-treated mice compared with controls. By comparison, the total number of miRNAs expressed in liver tissues from RDX-treated mice was lower than that in controls [see Supplemental Material, Table 1 (http://www.ehponline.org/members/2008/11841/suppl.pdf)]. In brain tissues, we detected 58 miRNAs in treated mice but not in controls, whereas we detected only four miRNAs (miR-10a, miR-10b, miR-712*, and miR-715) in control brain tissues but not in treated samples [see Supplemental Material, Table 3 (http://www.ehponline.org/members/2008/11841/suppl.pdf)]. Although many miRNAs were aberrantly expressed after RDX exposure, almost all detected miRNAs, either in control or in treated mice but not in both, gave low microarray signals, suggesting these miRNAs were expressed at low levels. Interestingly, of the total 191 miRNAs detected in one sample (either control or treatment) but not in both, one-third were miRNA* sequences. Usually, miRNA* sequences were quickly degraded during the final stage of miRNA biogenesis (Schwarz et al. 2003). What caused these miRNA* sequences to become detectable after exposure to RDX is unclear.

**Figure 4:**
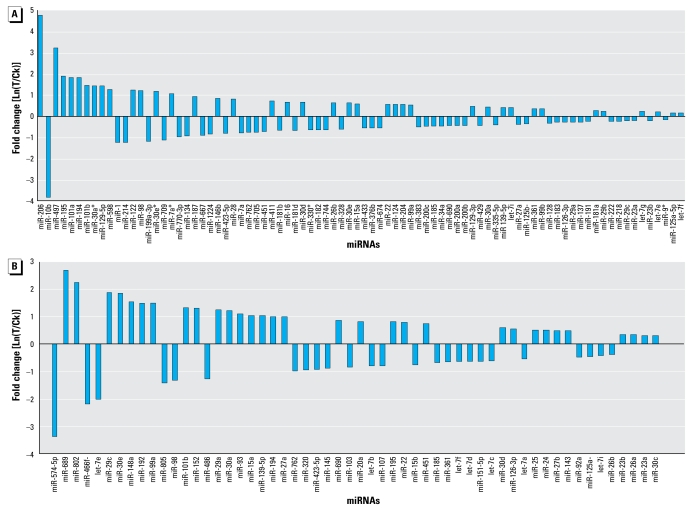
Fold changes of miRNAs whose expression levels were significantly altered in brain tissues (*A*) and liver tissues (*B*) of mice exposed to 5 mg/kg RDX for 28 days.

RDX exposure affected not only the total number of detectable miRNAs in mouse brain and liver, but also the expression levels of miRNAs (*p* < 0.01; Figure 3, Tables 1 and 2). The expression levels of 84 miRNAs were significantly altered in mouse brain after RDX exposure (Table 1, Figures 3, 4A). Of the 84 miRNAs, 38 were up-regulated and 46 were down-regulated. However, the extent of changes varied among miRNAs. For example, the expression levels of miR-206 and miR-497 were increased significantly by 26- and 9-fold, respectively, whereas the expression of miR-10b was decreased significantly by 14-fold (Table 1). Similarly, the changes in miRNA expression of liver tissues varied among miRNAs (Table 2, Figures 3, 4B). RDX exposure significantly affected the expression of 56 miRNAs in mouse liver (Table 2, Figures 3, 4B). Of these 56 miRNAs, 31 were up-regulated and 15 were down-regulated. The most up-regulated miRNAs were miR-689, miR-802, miR-29c, and miR-30e, and their expression levels were increased at least 3-fold. In contrast, the expressions of some miRNAs were significantly inhibited by RDX exposure; the miRNAs with at least 4-fold inhibition were miR-574-5p, miR-466f-3p, and let-7e.

Two major evidences suggest that brain was more sensitive to RDX exposure than liver. First, the number of miRNAs exhibiting expression alterations after RDX exposure is larger in brain tissues than in liver tissues. Eighty-four miRNAs have significant expression alterations in mouse brain, whereas only 56 miRNAs have significant expression alterations in the liver. Second, the changes in miRNA expression level were much higher and wider in the brain than in the liver. The fold change miRNA expression ranged from 14-fold down (miR-10b) to 26.5-fold up (miR-206) in the brain, compared with only 10-fold down (miR-574 -5p) to 6.5-fold up (miR-689) in the liver (Tables 1 and 2).

Of the 113 miRNAs with significantly aberrant expressions after RDX exposure, the expression levels of 10 miRNAs were significantly increased in both mouse liver and brain (*p* < 0.01): miR-99a, miR-30a, miR-30d, miR-30e, miR-22, miR-194, miR-195, miR-15a, miR-139-5p, and miR-101b. Three miRNAs (miR-762, miR-423-5p, and miR-185) had expression levels significantly decreased in both brain and liver (*p* < 0.01). For most miRNAs, their responses to RDX exposure were not consistent between brain and liver (Table 3). Seven miRNAs (let-7e, miR-98, miR-361, miR-26b, miR-125a-5p, let-7i, and let-7f) were significantly up-regulated in the brain but down-regulated in the liver after RDX exposure. In contrast, another seven miRNAs (miR-126-3p, miR-23b, miR-27a, miR-29a, miR-29c, miR-451, and miR-690) were significantly up-regulated in the liver but down-regulated in the brain. Although 37 miRNAs had significant expression changes in both brain and liver, 57 miRNAs had significant expression changes in the brain but not in the liver and 29 miRNAs had significant expression changes in the liver but not in the brain (Table 3).

### Confirmatory studies on differentially expressed miRNAs by qRT-PCR

**Figure 5:**
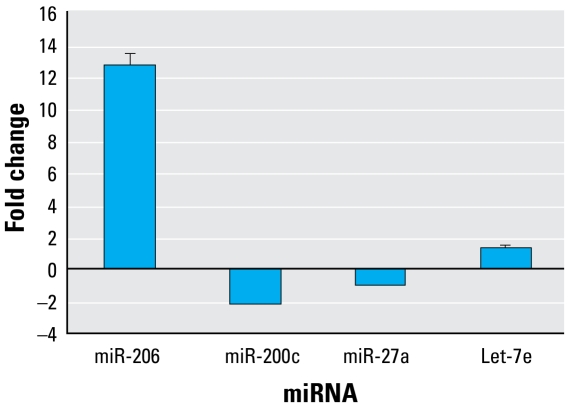
Confirmatory studies of selected miRNAs by TaqMan RT-PCR. The fold changes refer to the expression fold changes of the selected miRNAs in RDX-treated mice comparing with control mice. Values represent the mean ± SD of three independent samples, each run in triplicate.

To validate the microarray data, we assayed expression levels of four miRNAs (miR-206, miR-200c, miR-27a, and let-7e) by qRT-PCR and compared the results from the microarray and qRT-PCR. Of the miRNAs selected for comparison, two miRNAs (miR-206 and let-7e) were up-regulated whereas two miRNAs (miR-200c and miR-27a) were down-regulated based on the results of microarray analysis. The expression data obtained by qRT-PCR analysis are comparable with the microarray analysis data (Figure 5).

### Prediction of the target genes of miR-206

miRNAs function by targeting mRNAs for mRNA cleavage or translation repression. Thus, miRNA function studies depend heavily on the identification of miRNA targets. Currently, the major strategy to identify miRNA targets is based on various computational programs (Zhang et al. 2006). Almost all currently available computational programs overpredict miRNA targets. Several hundreds of genes have been predicted to be the targets of one single miRNA; only a few of them have been validated by experimental approaches, and most of these predicted targets are likely to be false targets (Zhang et al. 2006). One good approach to circumvent this problem is to employ two different computational programs to predict miRNA targets independently and then compare the two lists of targeted genes predicted by each program. The genes predicted by both programs are more likely to be the real targets of the miRNA.

In both microarray and qRT-PCR analyses, miR-206 was significantly up-regulated in mouse brain after exposure to RDX. The change of miR-206 expression was higher than all other miRNAs detected in this study. This suggests that miR-206 may play an important function in animal response to RDX exposure. To explore the potential functions of miR-206, we employed two computational programs (TargetScan and PicTar) to predict the targets of miR-206. After systematically analyzing two lists of miR-206–targeted genes predicted by TargetScan and PicTar, we identified the 50 most promising potential targeted genes [see Supplemental Material, Table 4 (http://www.ehponline.org/members/2008/11841/suppl.pdf)]. These candidate targets include several categories. First, these candidate targets contained several transcriptional factors (e.g., the zinc finger protein, the cAMP-responsive element binding protein, and the paired box gene). It is well known that miRNAs target transcriptional factors for regulating animal development. Second, stress-associated genes were predicted as targets of miR-206. Third, miR-206 may target brain-derived neurotrophic factors (BDNFs), which may cause neurologic disorders. Fourth, miR-206 may directly target cancer-related genes, for example, met proto-oncogene (NM_000245, GenBank) and v-ets erythroblastosis virus E26 oncogene homolog 1 (NM_005238, GenBank). This suggests that miR-206 may regulate multiple biological and metabolic processes in response to RDX exposure. Further investigating the functions of miR-206 on these potential targeted genes will help us better understand the mechanism of RDX-induced neurotoxicity and potential carcinogenesis.

## Discussion

### miRNA response to chemical exposure

We found the expressions of 113 miRNAs, belonging to 75 families, to be affected by RDX exposure in mice. Although a number of the RDX-responsive miRNAs (let-7, miR-34, miR-146, and miR-222) found in this study have been reported previously to have aberrant expressions in response to different chemical exposures in human cell lines, most of the RDX-responsive miRNAs reported in this study have not been shown to respond to chemical treatment previously (Blower et al. 2008; Marsit et al. 2006; Moffat et al. 2007; Pogribny et al. 2007; Rossi et al. 2007; Saito et al. 2006; Sun et al. 2008). These results suggest that either the spectrum of miRNAs that respond to chemical treatment is unique for specific groups of chemicals or that there are methodologic differences in detection of chemically responsive miRNAs. The investigation of the effect of different chemicals on miRNA expression profiles will allow a better understanding of the response spectrum of miRNAs in relation to chemical-induced toxicity and diseases. A particularly interesting potential application of these results is the use of miRNA response profile to develop a set of sensitive biomarkers for monitoring and assessing the health effects of environmental toxicants.

The research reported here possibly marks the first time that the effects of an environmental toxicant on the expression of miRNAs has been examined *in vivo*. Consequently, we are now in a position to compare these results with similar studies that have measured the response of miRNAs to pharmaceutical agents, which allows us to determine whether there are common miRNA response pathways for xenobiotic agents or whether the collective miRNA response is a function of specific chemical exposure. We will also be able to use the results of this research to understand the effects of environmental toxicants on environmental and human health.

This is also the first comparison study of miRNA expression in different tissues of mice exposed to an environmental toxicant. The contrasting expression of a same miRNA in brain relative to liver (some miRNAs were up-regulated in brain but down-regulated in liver and vice versa) indicates that there is tissue-specific variation in response to the same chemical and that miRNAs may play different roles in different tissues. We also determined that the response of brain miRNAs to RDX exposure was more pronounced than that of the liver, which is indicative that the toxic effects of RDX exposure may be more severe in the brain than in the liver (Figure 4). This further suggests that miRNAs respond to chemical exposure in a tissue-specific manner and that the toxicity may be mediated, at least in part, through miRNAs.

### RDX-induced regulation of miRNAs and their targets involved in neurotoxicity

It has been well known for some time that RDX exposure causes adverse central nervous system (CNS) syndromes, including convulsion, epileptic seizure, and loss of reflexes in human and experimental animals (Burdette et al. 1988; Goldberg et al. 1992; Levine et al. 1981; Pan et al. 2007; Testud et al. 1996). However, the molecular mechanism of RDX-induced neurotoxicity is unknown. In this study, we documented significant changes in miRNA expression in the brains of RDX-treated animals relative to their untreated controls. Of particular interest, miR-206 exhibited the most significant up-regulation (26-fold) in RDX-exposed mouse brain relative to controls. This result is notable because the *BDNF* gene is among the potential miR-206 targets. *BDNF* is one of the most important members of the neurotrophin family, and it is broadly expressed in mammalian (including human) CNS and peripheral nervous system and is thought to play important roles in supporting neuronal survival and differentiation, neurite outgrowth, and synaptic plasticity (Murer et al. 2001). Neurodegenerative diseases, including Alzheimer’s disease and Parkinson disease, have been associated with reduced *BDNF* expression (Murer et al. 2001). One recent study demonstrated that the environmental toxicant polybrominated diphenyl ethers reduced *BDNF* proteins in hippocampus of mice (Viberg et al. 2008), which suggests that neurotoxic environmental toxicants may exert their toxic effects through their activity on *BDNF* expression. In this present study, six transcript variants of *BDNF* genes are uniformly predicted to be the targets of miR-206 by both PicTar and TargetScan programs [see Supplemental Material, Table 4 (http://www.ehponline.org/members/2008/11841/suppl.pdf)] underscoring the likelihood that RDX-induced increases in expression of miR-206 may contribute to the neurotoxicity of RDX in the brain through its reduction of *BDNF* gene expression.

Although the regulation of the *BDNF* gene by various factors has been extensively investigated at the mRNA and protein levels (Murer et al. 2001), the role of miRNAs in *BDNF* gene regulation was only recently reported in a study that demonstrated that multiple differentially expressed miRNAs act as inhibitors of *BDNF* in human prefrontal cortex (Mellios et al. 2008). In that study, luciferase assays confirmed that *BDNF* was targeted by two miRNAs, miR-30a-5p and miR-195 (Mellios et al. 2008). Interestingly, we also found these two confirmed *BDNF*-targeting miRNAs (miR-30a and miR-195) were significantly up-regulated in mouse brain and liver after RDX exposure (Tables 1 and 2, Figure 4). In addition, two other members of the miR-30 family (miR-30d and miR-30e) that target *BDNF* (Mellios et al. 2008) were also overexpressed (Tables 1 and 2, Figure 4). This supports the role of *BDNF* as a potentially significant target gene of multiple miRNAs. Mellios et al. (2008) failed to detect miR-206 expression in human parietal cortex tissues using microarray analysis, although they predicted computationally that a target site for miR-206 exists in *BDNF* gene. However, both our microarray and qRT-PCR findings indicate that miR-206 was strongly up-regulated in brain tissues of mice exposed to RDX. These results suggests several alternative but not mutually exclusive hypotheses, including a regulatory interaction between miR-206 and *BDNF* that responds significantly to RDX treatment in brain tissues, or that miR-206 may be a stress response miRNA that only activates in response to specific toxicant exposure, including RDX.

### RDX-induced regulation of miRNAs and their targets involved in carcinogenesis

U.S. EPA has classified RDX as a class C potential human carcinogen (U.S. EPA 1988) based on a laboratory study in which RDX exposure elevated tumor incidence in B6C3F1 mice (Lish et al. 1984). However, the molecular mechanism of the RDX-induced carcinogenesis remains unknown. An interesting finding of this study is that a number of miRNAs with aberrant expressions after RDX exposure are also frequently deregulated in a wide range of cancers. Since the initial report linking miRNAs and cancer development (Calin et al. 2002), almost all types of cancers have been found to be associated with the aberrant expression of at least one miRNA, in which miRNAs function as oncogenes or tumor suppressor genes (Wiemer 2007; Zhang et al. 2007a). Importantly, over- or underexpression of one single miRNA resulted in tumor cell growth inhibition (Takamizawa et al. 2004) or tumor invasion and metastasis (Ma et al. 2007). For example, overexpression of miRNA let-7 significantly inhibited A549 lung cancer cell growth *in vitro* (Takamizawa et al. 2004), suggesting a potential novel approach for clinical cancer gene therapy by controlling the expression of a single miRNA (Zhang and Farwell 2008). In this study, we found that many cancer-related miRNAs, such as let-7, miR-17-92, miR-10b, 125b, miR-146, miR-15, miR-200, and miR-16, were significantly affected by RDX exposure (Table 4). Expression profiles of these miRNAs were significantly altered in many types of cancers, such as breast, lung, ovarian, liver, and prostate cancer, colorectal neoplasia, hepatocellular carcinoma, and chronic lymphocytic leukemia (Zhang et al. 2007a). This suggests that miRNAs may be involved in RDX-induced carcinogenesis.

In addition to the possibility that RDX-induced miRNAs are involved in carcinogenesis, we found that several potential miRNA-targeted genes are involved in cancer development. One example is the *TNKS-2* gene cluster (tankyrase 2, TRF1-interacting ankyrin-related ADP-ribose polymerase 2; NM_025235, GenBank), which is predicted to be a potential target of miR-206 [see Supplemental Material, Table 4 (http://www.ehponline.org/members/2008/11841/suppl.pdf)]. *TNKS-2* and its close homolog *TNKS-1* are positive regulators of telomere elongation, which is important for the perpetual growth of cancer cells (Cook et al. 2002). *TNKS* functions by interacting with telomeric repeat binding factor 1 (*TRF1*), which inhibits the telomerase activity to terminate telomere elongation. Binding of TRF1 to the telomere can be inhibited by ADP ribosylation of TRF1 by *TNKS-1* and *TNKS-2* (Cook et al. 2002; Smith et al. 1998). Aberrant expression of *TNKS* has been found in cancers and neoplasms, including multiple myeloma, plasma cell leukemia (Xu et al. 2001), bladder and colon cancer (Shebzukhov et al. 2008), and breast cancer (Sidorova et al. 2006). Knockdown of *TNKS-1* by small interfering RNA blocked mitosis due to sister chromatids remaining associated in telomeres (Dynek and Smith 2004). The inhibition of tankyrase and telomerase caused telomere shortening and apoptosis, which has been proposed as a potential cancer therapy (Seimiya 2006). Despite the association among *TRF-1* and *TNKS* and cell growth control, there have been no reports concerning miRNA-mediated regulation of *TNKS* gene clusters. We observed, using combined PicTar and TargetScan analyses, that *TNKS-2* was a target of miR-206, which implies that the strong up-regulation of miR-206 may target *TNKS-2*, which in turn may be a protective mechanism against the potential carcinogenic effects of RDX by inducing telomere elongation termination and apoptosis. This provides a new insight to elucidate carcinogenesis potentials of RDX.

**Figure 6:**
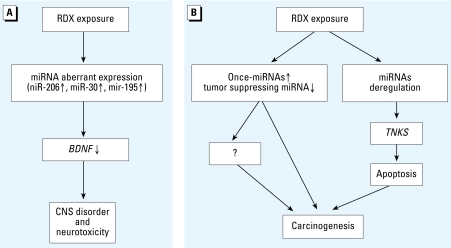
A proposed model showing the mechanisms of RDX-induced CNS disorder (*A*) and carcinogenesis (*B*). In this model, continuous lines show steps confirmed in this study or elsewhere; the dashed lines represent processes that have not been proved by direct evidence. RDX exposure caused aberrant expressions of many miRNAs. Some of them, for example, miR-106, miR-30, and miR-195, target *BDNF*, one of the most important members in the neurotrophin family that is widely expressed in mammalian CNS and peripheral nervous system (Murer et al. 2001; Wetmore et al. 1991). RDX-induced overexpression of miRNAs (miR-206, miR-30, and miR-195) inhibits the expression of *BDNF* gene, which contributes to neurotoxicity and CNS disorders. RDX exposure also induced aberrant expressions of onco-miRNAs and tumor-suppressing miRNAs that would directly or indirectly regulate tumor pathogenesis or target genes related to cell cycle (e.g., *TNKS*) to regulate cell apoptosis and cancer development. These proposed molecular changes, together with others, eventually cause RDX-induced carcinogenesis and CNS disorders. However, more studies need to be performed for testing this model.

Analyses of differentially expressed miRNAs and their targets in brain and liver tissues of mice exposed to RDX indicate that several miRNAs and their potential targets are involved in a complex regulatory network that encompasses a host of biological and metabolic processes. The RDX-induced miRNA-mediated changes in these processes have the potential to contribute to the RDX-induced heath effects, including neurotoxicity and potential carcinogenesis, two major concerns related to RDX exposure. miRNAs may directly and/or indirectly (through regulating other genes) affect the action of RDX on tumor pathogenesis and neurotoxicity (Figure 6). Recent studies on miRNA functions in carcinogenesis, cancer metastasis, and CNS disorders provide further support for this hypothesis. Further investigating the roles of miR-206 in regulation of *BDNF* and *TNKS-2* may lead to a novel miRNA-related gene therapy for the treatment of diseases associated with *BDNF* and *TNKS* gene expression. Also, the study of stress response miRNAs will facilitate the development of sensitive miRNA biomarkers for monitoring the health effects of environmental toxicants.

## Figures and Tables

**Table 1:** Eighty-four miRNAs with significant expression levels in mouse brain tissues after exposure to 5 mg/kg RDX for 28 days (*p* < 0.01).

miRNAs	Control (Ck) signal	Treatment (T) signal	Ln (T/Ck)	Fold change	miRNAs	Control (Ck) signal	Treatment (T) signal	Ln (T/Ck)	Fold change
Up-regulated (*n* = 38)					miR-29c	18243.37	15988.93	−0.22	0.86
miR-206	62.19	1603.19	4.73	26.53	miR-218	19242.65	16257.02	−0.22	0.86
miR-497	78.32	683.40	3.18	9.04	miR-222	7927.73	6898.47	−0.22	0.86
miR-195	5420.90	18586.65	1.85	3.60	miR-191	12497.48	10563.47	−0.24	0.85
miR-101a	414.29	1384.95	1.82	3.52	miR-137	7815.85	6503.00	−0.25	0.84
miR-194	197.96	672.73	1.80	3.49	miR-29a	35778.41	30011.86	−0.25	0.84
miR-101b	145.54	378.38	1.43	2.70	miR-126-3p	14784.18	12600.23	−0.27	0.83
miR-30a*	301.75	823.11	1.40	2.64	miR-183	11607.92	9357.58	−0.27	0.83
miR-129-5p	218.09	576.45	1.39	2.63	miR-128	44764.78	36552.18	−0.31	0.81
miR-598	392.37	957.26	1.22	2.34	miR-125b-5p	43002.58	33354.35	−0.35	0.78
miR-122	741.33	1683.98	1.20	2.30	miR-27a	4920.33	3500.16	−0.36	0.78
miR-98	1676.79	3775.53	1.19	2.28	miR-335-5p	5043.28	3912.21	−0.40	0.76
miR-30e*	250.04	540.47	1.16	2.23	miR-429	12415.46	9289.49	−0.42	0.75
miR-7a*	219.35	429.92	1.03	2.04	miR-200b	19737.09	14444.82	−0.44	0.74
miR-187	349.58	687.98	0.89	1.85	miR-200a	9521.66	6901.67	−0.45	0.73
miR-146b	362.94	611.32	0.82	1.77	miR-690	5227.26	3879.89	−0.45	0.73
miR-28	276.74	463.37	0.80	1.74	miR-34a	1717.74	1251.73	−0.45	0.73
miR-411	287.46	486.04	0.71	1.64	miR-185	4515.15	3236.95	−0.46	0.72
miR-16	15325.33	23835.23	0.65	1.57	miR-200c	10497.13	7489.56	−0.47	0.72
miR-30d	6457.60	10059.60	0.64	1.56	miR-383	1408.73	991.23	−0.50	0.71
miR-26b	8889.40	13087.02	0.61	1.52	miR-674	1832.33	1267.67	−0.54	0.69
miR-30e	2673.12	4166.48	0.60	1.52	miR-376b	1191.48	807.15	−0.55	0.69
miR-15a	1416.54	2199.71	0.57	1.48	miR-433	4293.85	2893.60	−0.55	0.68
miR-22	986.42	1449.72	0.53	1.45	miR-328	2162.73	1441.62	−0.61	0.66
miR-124	24353.80	34061.86	0.53	1.44	miR-744	1334.35	888.29	−0.62	0.65
miR-204	6615.88	9502.37	0.52	1.44	miR-182	9376.05	6027.11	−0.63	0.65
miR-99a	2198.46	3034.79	0.51	1.43	miR-330*	820.65	497.40	−0.64	0.64
miR-129-3p	5616.21	7610.62	0.43	1.35	miR-181d	970.01	600.68	−0.65	0.64
miR-30a	8398.62	11447.01	0.42	1.34	miR-181b	2470.77	1563.50	−0.67	0.63
miR-139-5p	8644.69	11096.41	0.40	1.32	miR-451	2271.21	1403.72	−0.71	0.61
let-7i	19829.87	24407.14	0.38	1.30	miR-705	1915.43	1168.91	−0.74	0.60
miR-361	5810.54	7223.99	0.34	1.26	miR-762	1214.54	727.44	−0.76	0.59
miR-99b	3474.25	4415.96	0.33	1.26	miR-7a	6007.06	3500.84	−0.78	0.58
miR-181a	8172.97	9590.44	0.24	1.18	miR-423-5p	1247.31	741.65	−0.80	0.57
miR-29b	6102.84	7130.04	0.23	1.17	miR-1224	1215.84	660.03	−0.84	0.56
let-7g	28189.65	32541.92	0.21	1.15	miR-667	2752.98	1448.71	−0.88	0.54
let-7e	24340.51	27969.73	0.18	1.13	miR-134	439.35	224.72	−0.91	0.53
miR-125a-5p	30571.05	33774.05	0.14	1.10	miR-770-3p	566.45	307.74	−0.98	0.51
let-7f	35296.63	39682.61	0.14	1.10	miR-709	65443.45	30144.97	−1.10	0.47
Down-regulated (*n* = 46)					miR-199a-3p	306.18	144.31	−1.17	0.44
miR-9*	24490.19	21896.30	−0.16	0.89	miR-214	258.41	108.21	−1.21	0.43
miR-23b	20805.98	18196.75	−0.20	0.87	miR-1	462.98	194.98	−1.22	0.43
miR-23a	17809.02	15248.49	−0.22	0.86	miR-10b	137.98	10.11	−3.80	0.07

**Table 2:** Fifty-six miRNAs with significant expression levels in mouse liver tissues after exposure to 5 mg/kg RDX for 28 days (*p* < 0.01).

miRNAs	Control (Ck) Signal	Treatment (T) Signal	Ln (T/Ck)	Fold change
Up-regulated (*n* = 31)				
miR-689	53.64	321.49	2.69	6.47
miR-802	102.06	452.31	2.25	4.76
miR-29c	650.72	2353.21	1.87	3.66
miR-30e	241.16	872.41	1.85	3.62
miR-148a	2798.83	8285.51	1.53	2.88
miR-192	8975.90	23801.68	1.49	2.82
miR-99a	62.76	182.85	1.48	2.79
miR-101b	271.45	691.85	1.32	2.49
miR-152	138.25	411.63	1.31	2.48
miR-29a	5693.53	13089.25	1.24	2.36
miR-30a	1231.17	2988.00	1.23	2.34
miR-93	85.17	186.80	1.10	2.14
miR-15a	493.75	978.07	1.04	2.06
miR-139-5p	129.23	295.12	1.03	2.05
miR-194	6147.52	12165.48	1.00	1.99
miR-27a	806.70	1598.30	0.99	1.98
miR-690	827.18	1477.09	0.86	1.82
miR-20a	344.54	589.10	0.80	1.75
miR-195	375.51	619.41	0.79	1.73
miR-22	900.37	1538.86	0.79	1.73
miR-451	885.93	1482.35	0.74	1.67
miR-30d	1156.83	1791.33	0.60	1.51
miR-126-3p	7922.97	11017.61	0.54	1.45
miR-25	557.96	796.59	0.51	1.42
miR-24	1582.59	2283.18	0.50	1.41
miR-27b	2272.75	3102.84	0.48	1.39
miR-143	667.41	1005.61	0.48	1.39
miR-23b	7607.74	9496.82	0.35	1.27
miR-26a	19218.42	23515.59	0.34	1.27
miR-23a	5561.59	6882.01	0.30	1.23
miR-30c	6790.49	8329.45	0.29	1.22
Down-regulated (*n* = 25)				
miR-26b	12561.71	9299.89	−0.38	0.77
let-7i	3777.32	2842.92	−0.42	0.75
miR-125a-5p	1505.59	1067.46	−0.47	0.72
miR-92a	3658.17	2635.02	−0.47	0.72
let-7a	31984.07	20972.51	−0.54	0.69
let-7c	29197.98	18108.06	−0.60	0.66
miR-151-5p	1583.62	967.48	−0.61	0.66
let-7d	27906.40	17206.77	−0.62	0.65
let-7f	31965.56	20730.37	−0.63	0.65
miR-361	1316.31	813.85	−0.66	0.63
miR-185	766.30	489.99	−0.67	0.63
miR-15b	1419.61	831.88	−0.75	0.60
miR-107	1044.56	607.84	−0.80	0.57
let-7b	24395.46	13082.76	−0.80	0.57
miR-103	1112.64	590.07	−0.83	0.56
miR-145	861.14	456.09	−0.88	0.54
miR-423-5p	640.50	338.97	−0.91	0.53
miR-320	1031.88	524.74	−0.95	0.52
miR-762	2142.91	1069.24	−0.98	0.51
miR-486	866.92	347.48	−1.27	0.42
miR-98	304.26	119.05	−1.32	0.40
miR-805	3046.89	1115.89	−1.42	0.37
let-7e	6778.31	1657.50	−2.01	0.25
miR-466f-3p	241.33	55.51	−2.17	0.22
miR-574-5p	1012.75	94.86	−3.37	0.10

**Table 3:** Comparison of miRNA expression profiles between mouse liver tissues and brain tissues after exposure to 5 mg/kg RDX for 28 days (*p* < 0.01).

	miRNAs	
miRNA category	No.	Name
Up-regulated in both brain and liver	10	miR-99a, miR-30e, miR-30d, miR-30a, miR-22, miR-195, miR-194, miR-15a, miR-139-5p, miR-101b
Down-regulated in both brain and liver	3	miR-762, miR-423-5p, miR-185
Up-regulated in brain but down-regulated in liver	7	let-7e, miR-98, miR-361, miR-26b, miR-125a-5p, let-7i, let-7f
Up-regulated in liver but down-regulated in brain	7	miR-126-3p, miR-23b, miR-27a, miR-29a, miR-29c, miR-451, miR-690
Significant expression changes in brain but not in liver	57	miR-99b, miR-1, miR-101a, miR-10b, miR-122, miR-1224, miR-124, miR-125b-5p, miR-128, miR-129-3p, miR-129-5p, miR-134, miR-137, miR-146b, miR-16, miR-181a, miR-181b, miR-181d, miR-182, miR-183, miR-187, miR-191, miR-199a-3p, miR-200a, miR-200b, miR-200c, miR-204, miR-206, miR-214, miR-218, miR-222, miR-23a, miR-28, miR-29b, miR-30a*, miR-30e*, miR-328, miR-330*, miR-335-5p, miR-34a, miR-376b, miR-383, miR-411, miR-429, miR-433, miR-497, miR-598, miR-667, miR-674, miR-705, miR-709, miR-744, miR-770-3p, miR-7a, miR-7a*, miR-9*, let-7g
Significant expression changes in liver but not in brain	29	let-7a, let-7b, let-7c, let-7d, miR-103, miR-107, miR-143, miR-145, miR-148a, miR-151-5p, miR-152, miR-15b, miR-192, miR-20a, miR-23a, miR-24, miR-25, miR-26a, miR-27b, miR-30c, miR-320, miR-466f-3p, miR-486, miR-574-5p, miR-689, miR-802, miR-805, miR-92a, miR-93

**Table 4:** Identification of currently known cancer-related miRNAs, whose expression levels were significantly altered after exposure to 5 mg/kg RDX for 28 days (*p* < 0.01).

miRNAs with significantly altered expression levels after exposure to RDX (*p* < 0.01)		
Brain	Liver	Cancers^a^
let-7**↑**	let-7**↓**	Lung cancer, prostate cancer, colorectal neoplasia
	miR-103**↓**	Stomach cancer
miR-10b**↓**		Breast cancer, colorectal neoplasia
miR-125a**↑**, miR-125b**↓**	miR-125a**↓**	Breast cancer, ovarian cancer, HCC
miR-128**↓**		Brain cancer (glioblastoma), prostate cancer
	miR-143**↑**	Colorectal neoplasia
	miR-145**↓**	Breast cancer, ovarian cancer, colorectal neoplasia
miR-146b**↑**		Breast cancer, papillary thyroid carcinoma
	miR-148a**↑**	Pancreatic cancer
miR-15a**↑**	miR-15a**↑**, miR-15b**↓**	CLL, pituitary adenomas
	miR-152**↑**	Pituitary adenomas
miR-16**↑**		CLL, pituitary adenomas
miR-181a**↑**, miR-181b,d**↓**		Brain cancer (glioblastoma), papillary thyroid carcinoma, pancreatic cancer
miR-183**↓**		Colorectal neoplasia
miR-191**↓**		Pituitary adenomas
	miR-192**↑**	Pituitary adenomas
miR-195**↑**	miR-195**↑**	Prostate cancer, HCC
miR-199a3p**↓**		Ovarian cancer
miR-200a,b,c**↓**		HCC, ovarian cancer, cholangiocarcinoma
	miR-20a**↑**	Colorectal neoplasia
miR-218**↓**		Stomach cancer
miR-222**↓**		Papillary thyroid carcinoma
	miR-24**↑**	Pituitary adenomas
miR-26b**↑**	miR-26a**↑**, miR-26b**↓**	Pituitary adenomas
miR-98**↑**	miR-98**↓**	Pituitary adenomas

**↑**, up-regulation; **↓**, down-regulation.

a Abbreviations: CLL, chronic lymphocytic leukemia; HCC, hepatocellular carcinoma.
